# Compositional Mining of Multiple Object API Protocols through State Abstraction

**DOI:** 10.1155/2013/171647

**Published:** 2013-06-03

**Authors:** Ziying Dai, Xiaoguang Mao, Yan Lei, Yuhua Qi, Rui Wang, Bin Gu

**Affiliations:** ^1^School of Computer, National University of Defense Technology, Changsha 410073, China; ^2^Beijing Institute of Control Engineering, Beijing 100190, China

## Abstract

API protocols specify correct sequences of method invocations. Despite their usefulness, API protocols are often unavailable in practice because writing them is cumbersome and error prone. Multiple object API protocols are more expressive than single object API protocols. However, the huge number of objects of typical object-oriented programs poses a major challenge to the automatic mining of multiple object API protocols: besides maintaining scalability, it is important to capture various object interactions. Current approaches utilize various heuristics to focus on small sets of methods. In this paper, we present a general, scalable, multiple object API protocols mining approach that can capture all object interactions. Our approach uses abstract field values to label object states during the mining process. We first mine single object typestates as finite state automata whose transitions are annotated with states of interacting objects before and after the execution of the corresponding method and then construct multiple object API protocols by composing these annotated single object typestates. We implement our approach for Java and evaluate it through a series of experiments.

## 1. Introduction

In object-oriented programs, programmers write code by invoking various application programming interfaces (APIs). In general, not all method invocation sequences are legal. There are constraints on the temporal order of invocations of related methods. For example, programmers should not *write* into a file after it has been *closed*. *API protocols* specify which API method call sequences are allowed. API protocols are very useful in many software engineering activities. They can aid the generation of test cases [1]. Program verification tools can use API protocols as input to prove the absence of protocol violations [2, 3], and program analysis tools can use them to find certain errors [4–7]. In addition, formal specifications including temporal API specifications can support the understanding of correct software behavior [8], which is central to software maintenance.

As writing API protocols is cumbersome and requires expert knowledge of corresponding APIs, they are often missing, incomplete, or out of date despite their usefulness. To address this problem, researchers have developed specification mining techniques to mine API protocols from API client programs [9–17]. Many existing approaches focus on API protocols of single objects [14–16]. However, an object is not isolated; they interact by invoking each other's methods. Single object protocols are too restrictive because some API protocols can only be expressed by specifying multiple interacting objects. For instance, we must consider a collection and its iterator together to specify one of their safety properties that the contents of the collection should not be modified while its iterator is being used. Experiments in previous work [6] show that 41% of the detected issues can be only found with multiple object protocols.

According to the *information hiding* principle of object-oriented software engineering, states of an object should only be accessed and modified through the methods defined in this object's interface. Since objects interact by invoking each other's methods, the receiver object of a method invocation typically interacts with the method's parameter objects and return object if any. Moreover, objects can transitively affect other objects' behavior. As methods typically receive parameters as input and produce a return, object interactions are common. There are possible hundreds of millions of objects during the execution of realistic programs. For dynamic analysis approaches, the input trace data is usually very large (e.g., more than 240 million runtime events [10] and more than 98 million runtime events [11]). These objects compose a large and complex *interaction net*, which poses a major challenge to the mining of multiple object API protocols. On the one hand, we should consider all object interactions to mine precise and complete specifications. On the other hand, large sets of interacting objects lead to very high computational overhead that compromises the usefulness of the specification mining approach.

Typestates [18] are intended for capturing API protocols. The observation behind typestates is that whether an operation is available on an object depends not only on the type of the object but also its internal states. Researchers develop several typestate systems for object-oriented programs [19, 20]. State abstraction techniques to mine typestates based on explicit object states [14, 15] have been proven effective to mine useful API protocols of single objects. The main idea of these techniques is to use abstract values of object fields (or returns of the *observer* methods) to label states during the mining process. In this paper, we apply the idea of state abstraction to mine API protocols of multiple interacting objects. Our insight is that by labelled object states, we can conveniently identify the order of method invocations from different objects. We give a clear definition of *object interactions* based on the type definitions of objects, and it can capture all interacting objects. Based on this definition, our miner first mines single object typestates as finite state machines (FSMs) whose states are labelled by abstract field values and whose transitions are labelled with explicit states of interacting objects before and after the execution of the corresponding methods. Second, our miner extracts the typestates of the declared types (maybe concrete super types of the runtime types of objects or even abstract types) of parameters and returns of methods from the typestates of their implementing subtypes. Then, our miner products typestate FSMs of interacting objects without violations of the interacting constraints annotated with transitions of single object typestates. At last, state labels are discarded, and we get multiple object API protocols. The most important feature of our miner is that each object is mined separately without considering methods of other objects, which guarantees the scalability of our approach. The naive product of the typestate FSMs of different objects cannot capture the constraints of object interactions because the product allows arbitrary interleavings of method invocations from different objects.

Previous work on mining multiple object API protocols employs various approaches to cope with this challenge [10, 11, 13, 17, 21, 22]. In order to reduce the complexity of the analysis of object interactions, they utilize various heuristics to focus on small sets of related objects and methods and then mine subtraces of these related events by commonly used specification inference techniques. Pradel and Gross [10] present the method-centric approach that runtime events issued during a method's execution are assumed to be related to each other. Nguyen et al. [22] also confines interacting objects to the source code of a single method. Lee et al. [11] propose the event specification approach that methods involved in a unit test run are assumed to be interacting with each other. Yang et al. [17] and Nguyen et al. [21] utilize the predefined small specification templates such as the alternating pattern over event pairs to mine simple patterns dynamically and statically, respectively. Gabel and Su [13] first mine small patterns based on the predefined simple templates and then use inference rules to compose them to construct complex properties. These approaches are shown to be able to mine useful protocols. However, because the potential interactions with an object are determined by the type definition (e.g., signatures of methods) of the object, there may be some unpreferred object interactions that are filtered out. These approaches exchange some object interactions for the scalability. In contrast, our approach can capture all object interactions that manifest during runtime and scalably mine arbitrarily complex multiple object API protocols by composing the typestates of single objects.

The rest of this paper is organized as follows.  Section 2 introduces the background of object-oriented typestate systems and discusses their drawbacks when they are used to formalize API protocols.  Section 3 discusses our approach to mining multiple object API protocols by composing typestates of single objects.  Section 4 describes our implementation for Java and presents the experimental evaluation of our approach.  Section 5 discusses related work, and Section 6 concludes.

## 2. Background: Object-Oriented Typestates

The formalism of multiple object API protocols mined in this paper is inspired by the object specifications of several existing object-oriented typestate systems [19, 20]. Because typestates reflect how state changes of objects can affect valid method invocations, a typestate is an abstraction over concrete object states and can be characterized by the values of all fields of an object. Typestates are mapped onto the fields of the implementing class by defining a predicate for each typestate, called a *state invariant*, which can be any boolean combination of state tests, state comparisons, integer comparisons, boolean constants and fields. The substitutability of subtypes for super types is preserved by the *state refinement* that a subtype can define a set of substates as special cases of an existing state. The specification of a method can be changed through the *method refinement*. A method can be respecified more precisely in a subtype based on the refined substates. The main role of typestates is to specify methods. Equation (1) gives a simplified method specification language for typestates of object-oriented programs:

(1) $$\begin{array}{c} M∶=C\;|\;M\wedge M,\\ C∶=V\to V,\\ V∶=\left(s_{1},\ldots ,s_{n}\right). \end{array}$$

A method is specified with an intersection of cases, which means that all these cases hold. A *case* represents a state transition which is denoted as *A* → *B* to express that a method requires a source state *A* and produces a destination state *B*. The source state is a vector consisting of the states of the receiver of the method and its arguments (in their order in the signature). The destination state has one more state for the method's return object if any. Nondeterminism of state transitions can be expressed using the intersections of different cases. For example, *A* → *B*∧*A* → *C* represents that starting at state *A*, executions of a method can transition to state *B* or state *C*. The state invariant is evaluated to test whether an object is in a particular state. Either statically checked [19] or dynamically checked [20], state invariants of typestates are evaluated for every method invocation: source state violations are flagged as precondition violations, and destination state violations are flagged as postcondition violations. Source states and destination states are actually treated as preconditions and postconditions of corresponding methods, respectively.

These typestate systems have been proven useful for modeling protocols in object-oriented programs [19, 20]. However, there are mainly two drawbacks of them to specify API protocols. First, it is not trivial to derive state invariants as this requires expert knowledge of underlying classes. If there is any, semantic information of APIs is mainly within informal documents and needs to be manually extracted. Second, because typestates are tagged with state invariants which rely on values of fields of corresponding classes, these typestate systems cannot specify abstract types, such as interfaces and abstract classes in Java. Abstract types usually represent high-level abstractions and obey many common and important properties. Specifications of abstract types are more clear, explicit, and compact than that of their implementing classes.

## 3. Technical Approach

This section discusses the details of our approach. In Section 3.1, we define several concepts to formalize the idea of typestates composition. In Section 3.2, we present how to mine single object typestates annotated with object interactions through state abstraction. In Section 3.3, we present the technique to extract typestates for super types from typestates of their implementing subclasses. In Section 3.4, we discuss how to compose single object typestates into API protocols of multiple interacting objects.

**Figure 1:**
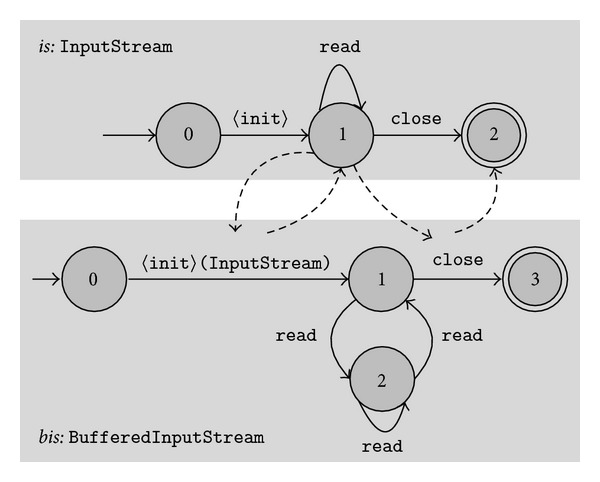
Typestates of interactions between BufferedInputStream and its wrapped InputStream mined by our approach. *is* denotes the above highlighted part of the figure, and *bis* denotes the below highlighted part of the figure.

Figure 1 shows the typestates of interactions between BufferedInputStream and its wrapped InputStream mined by our approach. This is the running example throughout our paper. All classes presented as examples in this paper are from the standard APIs of the Java language except explicitly stated. These typestates capture the *resource-wrapping protocol* that closing the wrapping resource will implicitly close the wrapped resource, so the wrapped resource can not be used any more after its wrapping resource is closed. The *is* part of this figure is the typestates of InputStream and the *bis* part is that of BufferedInputStream. Because InputStream is an abstract class, we obtain its typestates by extracting submodels from typestates of its implementing type through a state-preserving submodel extraction algorithm. Directed dashed lines represent interactions between these two typestate models. The directed dashed line from state 1 of *is* to <init> of *bis* denotes that an InputStream object should be in its state 1 before passed into <init> of BufferedInputStream as the parameter. This dashed line characterizes the common usage that the <init> of BufferedInputStream follows <init> of InputStream. The directed dashed line from close of *bis* to state 2 of *is* denotes that after the execution of close of BufferedInputStream, the wrapped InputStream is in its final state 2. This dashed line specifies the safety property that the wrapped InputStream cannot be used any more after the close of its wrapping BufferedInputStream.

### 3.1. Approach Overview

Here we give a high-level overview of our mining approach.

**Definition 1 (trace)**

A *trace T* = 〈*e*_1_,…, *e*_*n*_〉 is a sequence of events, where an *event e* = (*s*_1_, *m*, *s*_2_) is a triple, with *m* is the method execution, *s*_1_ is the state of the training program just before *m* enters, and *s*_2_ is the state of the training program just after *m* exits. For object-oriented programs, the program state is typically a set of objects each of which consists of a set of field-value pairs. We write *s*.*o* as the state of *o* when *s* denotes the state of the program.

**Definition 2 (interaction specification)**

Suppose *t* is a reference type (classes or interfaces in Java, excluding arrays). For every public method *t*_*r*_ *m*(*t*_1_,…, *t*_*n*_) of *T*, where *m* is the method name, *t*_*r*_ is the return type, and *t*_1_,…, *t*_*n*_ are parameter types, we omit *void* and primitive types of parameters and return and only keep reference types. The *interaction specification S*_*t*_ of *t* is the set *M* of all its public methods with retained reference parameters and returns. We use *P*_*t*_ to denote all retained parameters and returns in *S*_*t*_.

The interaction specification of a type is determined by its definition. We neither make assumptions nor employ heuristics. This is the power of our approach that it has the potential to capture all objects interacting with an object. For example, the interaction specification of BufferedInputStream is the set {<init> (InputStream), <init> (InputStream, _)} with _ as the place holder to indicate the position of each parameter. Methods that have no reference parameters and no reference return are omitted.

**Definition 3 (interacting objects)**

During runtime, if a method is invoked, its parameters and return of reference type if any are bound to *null* or specific objects. At some point during runtime, for an object *o* of reference type *t*, its *interacting objects* (objects interacting with it) form a set *O*_*o*_ that includes all objects that are bound to the parameters and returns of the methods in the interaction specification of *t*. We define the function *b* : *P*_*t*_ → *O*_*o*_ ∪ {*null*} to manifest the mapping between the interaction specification and the interacting objects. *b* involves as the program runs.

For an object, its interacting objects involve as the program runs. When a method is invoked at the first time for this object, the parameter objects and return object if any are added to *O*. If a method is invoked a second time, new bound objects are added to *O*, and old objects bound to the same parameter or return are replaced. The concept of interacting objects reflects one fact of dynamic analysis that object interactions that we can mine are limited to observed executions of underlying programs. Please note that the number of interacting objects in *O* for an object will not exceed the number of parameters and returns of all methods in the interaction specification of the type of this object. For example, when the method BufferedInputStream. <Init> enters, the interacting objects of its receiver includes only one object that is bound to its parameter InputStream.

**Definition 4 (multiple object API protocols)**

*Typestates annotated with interactions* for a reference type *t* are a nondeterministic finite state machine (NDFSA) *M* with transition annotations that *M* = (*Q*, Σ, *δ*, *λ*, *S*, *F*), where *Q* is a finite set of states that represent abstract object states, Σ is the alphabet that consists of the methods in the interaction specification of *t*, *δ* is the transition relation that is a subset of *Q* × Σ × *Q*, *λ* : *δ* → (*P*_*t*_ → *C*) is the annotation function that determines the state change for each interacting object and transition in *δ*, where *C* is the set of state changes. A *state change* of one interacting object represents that the state of this object changes from one to another, which can be denoted as *s*_1_ → *s*_2_ to express that the execution of the corresponding method associated with this transition requires a source state *s*_1_ and produces a destination state *s*_2_. *S* is the set of start states, and *F* is the set of final states. *Multiple object API protocols* for a set of objects are the set of typestates annotated with interactions, among which typestates of interacting objects are composed. By *composed*, we mean that all states in the interaction annotations are mapped to corresponding states in typestates of an type.

Figure 1 presents such an example of multiple object API protocols.

**Figure 2:**
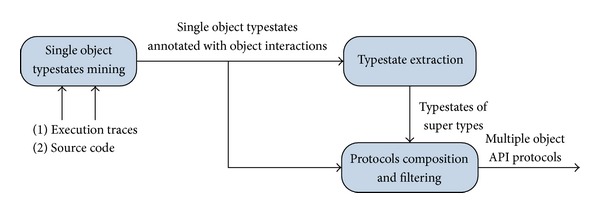
The architecture of our approach.

Figure 2 depicts the architecture of our approach. We take two types of inputs: the first is the source code of the target APIs, that is, for the identification of interaction specifications. The second are program execution traces with recorded values of object fields. We first mine single object typestates through state abstraction. These typestates are also annotated with abstract states of interacting objects to record object interactions. We then extract the typestates for super types from typestates of their implementing subclasses. At the last step, different typestates are composed together to get the API protocols of multiple interacting objects.

### 3.2. Mining Single Object Typestates Annotated with Interactions

We adopt the state abstraction technique to mine single object typestates and object interactions. To produce succinct and general models, abstract field values instead of concrete ones are used to label states. We use the same state abstraction function *abs* as [15], which is as follows: values of reference fields (objects or arrays) are abstracted to *null* (=*null*) or *not null* (≠*null*), values of numerical fields are abstracted to *larger than zero* (>0), *less than zero* (<0), or *equal to zero* (=0), and values of boolean fields remain unchanged. This state abstraction approach has been proved successful in mining single object typestate models [14, 15].  Algorithm 1 presents the algorithm to mine typestates of an object with interaction annotation. We define the function *f* : *δ* → (*P*_*t*_ → *℘*(*C*)) to record all observed state changes of objects bound to a parameter or return for a transition from the beginning of the program execution to now. *℘*(*C*) denotes the power set of *C*. Each state change is associated with a *frequency,* that is, the number of times this state change is observed. For each event of the object *o*, we determine abstract states of *o* and all its interacting objects just before and after the invocation. We get a transition of the method that goes from the source state to the destination state of *o* and add it to the model *M*. We annotate this transition with state changes of all interacting objects of *o*. When the algorithm runs to the end of the trace, we determine the annotation function *λ* by choosing the most frequent state transition (MAX(*C*)) and discarding others. The typestates of a concrete class consists of the union of all states and transitions of the typestates of all its objects. The annotation function has the value of the most frequent state change for each transition and parameter or return. The approach to get typestates of a super type is discussed in Section 3.3.

A state of interacting objects within the interaction annotations is associated with a parameter or return of the method in the interaction specification. If the declared type of the parameter or return is different from the runtime type of the interacting object bound to it, we also associate this interacting object with this state. This association is requisite for later typestates composition because different implementations of a type do not necessarily have the same fields. The typestates of single objects are mined in the per object way, which is essential to make our approach scalable. The time complexity of the algorithm in Algorithm 1 is determined by the length of the trace and the complexity of the interaction specification. If the trace contains *m* events and the interaction specification has *n* parameters and returns of all its methods, the complexity of the algorithm is *O*(*m* × *n*).

### 3.3. Extracting Typestates for Super Types

Abstract types such as interfaces and abstract classes in Java and the inheritance are common in object-oriented programs. The behavior of a super type can be manifested by objects of its implementing subclasses. However, except public methods declared in the super type, its implementing subclass usually has additional public methods. These additional methods are either specific to the implementing class or belong to another super type that the class implements simultaneously. The declared types of the parameters and returns of the methods in the interaction specifications may be abstract or super types of the type of the bound interacting objects. To get the multiple object API protocols of the interaction specifications, the additional methods that do not belong to the declared types must be removed from the typestates of the interacting objects. Moreover, to enable the composition of the typestates of single objects, states in the original typestates must be preserved in the result typestates with the additional methods removed. Existing FSM transformation algorithms [23] based on the accepted languages are not applicable here. In this section, we design an algorithm to extract the typestates of a super type from the typestates of its implementing subclasses, and meanwhile the states in the original typestates are preserved.

We first formalize the problem. Assume that the typestates of a super type are *M*_*s*_ = (*Q*_*s*_, Σ_*s*_, *δ*_*s*_, *λ*_*s*_, *S*_*s*_, *F*_*s*_), and the typestates of one of its implementing subclass are *M* = (*Q*, Σ, *δ*, *λ*, *S*, *F*) and Σ_*s*_⊆Σ. We define the typestate extraction function te: Σ* → Σ_s_* as follows: (1) te(*a*) = *a*, if *a* ∈ Σ_*s*_; (2) te(*a*) = *ε*, if *a* ∉ Σ_*s*_; (3) te(*ω*_1_ *ω*_2_) = te(*ω*_1_)te(*ω*_2_). Intuitively, the function te transforms a string into a new one that preserves only the interesting symbols in their original order. Based on te, we can formalize the typestates extraction problem as how to compute *M*_*s*_ from *M*, while *L*(*M*_*s*_) = {*ω* | ∃ *ω*′ ∈ *L*(*M*), s.t. *ω* = te(*ω*′)} and *Q*_*s*_⊆*Q* hold.

To solve this problem, we introduce the closure function cl: *Q* → *℘*(*Q*). The function cl⁡ maps a state to a set of states that are reachable from this state by following zero or more transitions with uninteresting input symbols. Formally, we define cl as follows: (1) for all *q* ∈ *Q*, *q* ∈ cl⁡(*q*); (2) for all *p* ∈ cl⁡(*q*)  ∧ for all *a* ∈ Σ − Σ_*s*_, *δ*(*p*, *a*) ∈ cl⁡(*q*). Now, we define the extended closure function ecl as follows:

(2) $$\begin{array}{c} \text{ecl}\left(Q^{\prime }\right)=\underset{q\in Q^{\prime }}{⋃}\mathrm{cl}\left(q\right),\;Q^{\prime }\subseteq Q. \end{array}$$

Based on cl and ecl, we formally specify *M*_*s*_ = (*Q*_*s*_, Σ_*s*_, *δ*_*s*_, *λ*_*s*_, *S*_*s*_, *F*_*s*_) where *Q*_*s*_⊆*Q*, *δ*_*s*_(*q*, *a*) = ecl(*δ*(*q*, *a*)), *λ*_*s*_ = *λ*↾_Σ_*s*__, that is, the restriction of *λ* to Σ_*s*_, *S*_*s*_ = ecl(*S*) and *F*_*s*_ = {*q* | cl⁡(*q*)∩*F* ≠ *∅*}. The most important feature of *M*_*s*_ is *Q*_*s*_⊆*Q*. The algorithm to solve this problem is presented in Algorithm 2. For each transition (*q*, *m*, *q*′) of *M* with *m* ∈ Σ_*s*_, compute cl⁡(*q*′). For every state *p* ∈ cl⁡(*q*′), add a transition (*q*, *m*, *p*) to *M*_*s*_. The worst case complexity of this algorithm is *O*(*m* ^2^ × *n* ^2^), where *m* is the number of states of the typestates and *n* is the number of transitions of the typestates. The complexity of this algorithm is high; however, we expect no high overhead in practice as typical typestates FSMs are small with a few tens of states and transitions.

If there are multiple implementing classes for a super type, we simply union typestates extracted from them. Such subtypestates are separate from each other, and we call them *typestates parts*. Every typestates part is tagged by the type of the implementing class where it is extracted. Simple union may produce large typestates, but we appreciate the merit that all states of different typestate parts are preserved from their implementing classes. Formally, if there are *n* implementing classes of a super type and the *n* extracted typstates parts are *M*_1_ = (*Q*_1_, Σ, *δ*_1_, *λ*_1_, *S*_1_, *F*_1_),…, *M*_*n*_ = (*Q*_*n*_, Σ, *δ*_*n*_, *λ*_*n*_, *S*_*n*_, *F*_*n*_), we define the protocol of the abstract type *M* = (*Q*, Σ, *δ*, *λ*, *S*, *F*), where

(3) $$\begin{array}{c} Q=\overset{n}{\underset{i=1}{⋃}}Q_{i},\;\;\delta =\overset{n}{\underset{i=1}{⋃}}\delta_{i},\;\;\lambda =\overset{n}{\underset{i=1}{⋃}}\lambda_{i},\\ S=\overset{n}{\underset{i=1}{⋃}}S_{i},\;\;F=\overset{n}{\underset{i=1}{⋃}}F_{i}. \end{array}$$

### 3.4. Typestates Composition and Filtering

We generate multiple object typestates by composing typestates of single objects. We perform the composition by finding the same state in corresponding typestates for every state in the interaction annotation. An algorithm to do this is presented in Algorithm 3. For a state *s* associated with an argument or return *p* in the interaction annotation of a transition, we try to identify the state *s*′ in the typestates of the declaring type *t* of *p* that has the same field-value label as *s*. If *t* is a concrete class, *s*′ is in the states of the typestates of this concrete class. If *t* is an abstract type, *s*′ is in the states of the typestate part of the typestates of this abstract type, which is extracted from the typestates of the object associated with *s*′. After finding the same state for every state in interaction annotations of all typestates, we discard the labels of states and identify them by abstract names such as numbers. We assure that within typestates of a type, states with different field-value labels have different names.

Because we discard the state labels, the final multiple object typestates specify the proper order of method executions. To check the behavior of a single object, the legal method execution sequences are the strings accepted by the typestates of the type of the object without considering the interaction annotations. The ordering constraints of method executions from different objects are imposed by the interaction annotation of typestate transitions. To check multiple object typestates, for a transition with method *m*, all state changes in its interaction annotation must be validated. To validate a state change, for every argument *p* of *m*, the method called on *p* immediately before *m* enters must be one of the methods directly reaching the source state of *p* in the state change, and the method called on *p* immediately after *m* exits must be one of the methods directly leaving the destination state of *p* in the state change. If there is any return object of *m*, the method executed on it immediately after *m* exits must be one of the methods directly leaving its state in the state change. Method executions from different objects can be arbitrarily interleaved if there are not direct or indirect constraints from the interaction annotations between them.

During typestate composition, we apply several rules to filter out uninteresting interactions. These uninteresting interactions stem from the common knowledge of software designs and limitations of the approach to mine single-object typestates through state abstraction. The latter case will be further discussed in Section 4.3. The first rule we utilize is the *package-based filtering* that is commonly used in multiple object API protocol mining approaches [4, 10, 11]. The rule assumes that objects from different packages are not likely to obey some common API protocols. Adhering to this rule, we only compose typestates of types from the same package. The second rule we utilize is that typestates with only one state are not considered. Typical one-state typestates include typestates for immutable objects such as strings, class wrappers, and classes without fields. One-state typestats cannot specify any method invocation orders. The last rule is that a state change is discarded if (1) this change has the same source and destination state, and (2) the object corresponding to this change is neither a parameter nor the return of the method of transition associated with this change, and (3) the transition associated with this change does not go into a final state. This rule is important to filter out many uninteresting interactions based on the observation that if the destination state of a state change does not change from the source state, the corresponding two objects often do not interact with each other. The condition (3) is to preserve interactions that the cleanup of an object usually implicitly cleans up its interacting objects. For example, during the mining of the typestates in Figure 1, the last rule filters out interaction annotations of read of BufferedInputStream but preserves the interaction annotation of close of BufferedInputStream.

## 4. Implementation and Results

 In this section, we describe the implementation and empirical evaluation of our approach. we also discuss several limitations of our current implementation.

### 4.1. Implementation

To obtain information required to mine typestates, We must *trace* program executions. For this purpose, we write an agent using Java Virtual Machine Tool Interface (JVMTI) [24]. JVMTI is convenient to trace programs in many aspects such as that it is easy to access the call stack and that we can attach a unique tag to every object. For both single-threaded and multithreaded applications, events are recorded in the order of their occurrence, that is, the order of events is preserved globally. In this way, object interactions with events coming from different threads can be recognized. The agent is attached to Java Virtual Machine and writes the flow of events to plain text files. To mine typestates, we need both information of method executions and information of object states. The tracing agent records three types of events: *Method Entry*, *Method Exit,* and *Field Modification*. A *Field Modification* event is issued when some value is assigned to a field of an object.  Table 1 presents the event types and recorded information for all events handled by the agent. The largest file we analyzed is about 2.2 GB in size and contains more than 106 million runtime events.

For a *Method Entry* event of a constructor, we create a State object to represent the state of the created object. All fields of the object have default values of the Java language. When a *Field Modification* event on this object is encountered, we update the corresponding field with the new value in the State object. The *Field Modification* event also captures the initialization of a field at its declaration. In this way, the State object maintains the state of the corresponding object. The object state maintained in the State object is used to extract abstract field values during typestates mining.

We can configure events of what types are to be traced, for example, by providing a package name to indicate that the tracing agent will record events of all public types in this package. Because we aim to mine API protocols, only *Method Entry* and *Method Exit* events of public instance methods are traced. The interaction specifications of types and other type information are obtained using Java's reflection utilities. We need access to the bytecode of target types. However, source code is not necessary. Our tracing agent is based on JVMTI that allows a much less complex and thus less error-prone implementation of the tracer. The downside of this approach is that the tracing agent incurs significant runtime overhead. However, our general approach is modular and is not bound to this tracing agent. Any traces that contain method executions with parameter and return values and states of involving objects can be fed into our typestates miner.

### 4.2. Empirical Evaluation

This section describes the experiments of applying our approach to several benchmarks from the literature. At first, we give the experimental setup and an overview of the benchmarks. Second, we show that object interactions are common by analyzing the interaction specifications of target APIs and present the mined typestates. Third, we evaluate the quality of mined typestates by examining whether they characterize typical APIs usages. For this aspect, we compare typestates for the same type mined from different applications. Finally, we discuss several typestate models automatically mined by our approach.

We apply our approach to mine typestates of types from three packages and their subpackages of Oracle Java JDK 6: java.lang, java.util and java.io, totally 17 packages. APIs in these packages obey important properties and are widely used as experimental targets in the literature [10, 11]. Training programs in our experiments are benchmarks from the DaCapo benchmark suite 2006-10-MR2, which ensures a controlled and reproducible execution of all benchmarks [25]. We use the tracing agent to record events into a plain text file for each of these benchmarks. We limit the execution time of every program to half an hour. Although programs do not run to its end for tracing, the result traces contain large enough numbers of events for our experimental evaluation.  Table 2 presents the traces used in our experiments. The elapsed execution time for two separate stages, namely, single object typestates mining (Section 3.2) and typestates extraction (Section 3.3), is also presented in Table 2. The time for typestates composition is not presented. Because we assign every state a unique number as its abstract name and record it in state changes during the process of mining single object typestates annotated with interactions, much of the work of typestates composition is saved. As our approach framework is modular, we present the algorithm for typestate composition in Algorithm 3 for potential use when other state labelling techniques or alternative implementations are used. The time used to mine typestates of single objects is roughly linear to the number of events in the input trace. The total time of the typestates mining is typically less than 10 minutes for a benchmark program. It is low considering the huge number of input events and is fast than recent work in the literature [10, 11]. Although having the complexity of *O*(*m* × *n*), where *m* is the number of states of the typestates and *n* is the number of transitions of the typestates, extracting typestates for super types is fast, typically in several minutes, because common single object typestates have a very small number of states and transitions.

Interaction specifications specify object interactions that potentially occur during runtime. Because the interaction specification of a type is determined by the type's definition (or structure), we present the statistics of object interactions collected from interaction specifications of public types in the target packages in Table 3. An *interaction* in this table is a pair of different types 〈*t*_1_, *t*_2_〉 that *t*_2_ is the type of a specific parameter of or that of the return of a specific public method of *t*_1_. They provide the background for evaluating multiple object typestates mining approaches. The data is obtained by analyzing the bytecode of target types with the Java reflection utilities. In accordance with the definition of the interaction specification, we only care for public types and public methods here. It can be seen that java.lang is more complex in terms of objects interactions with an average of 9.4 interactions per type. The least complex package is java.io that still has an average of 1.7 interactions per type. These indicate that common types will interact with more than one other type, and object interactions are common among APIs. In addition, there are a nontrivial number of types that potentially interact with many other types simultaneously. For example, in the package java.lang, there are 14 types that have no less than 10 interactions. Object interactions are not only common but also complex. During inspecting interaction specifications of these types, we also find that there are no simple indicators of which interaction being more likely to obey common usage protocols than others. So it is important to capture as many as possible object interactions to mine precise and complete typestates. Although the *package-based filtering* has been proved useful in practice, it is not easy to distinguish methods of the same type in terms of their protocol-obeying likeliness. The results of mined typestates are presented in Table 4. Compared with Table 3, It can be seen that there is a large part of types and interactions that are not covered by the mined typestates. However, this is due to the training programs that use only part of the target APIs. Our mining approach can capture all object interactions. we mine more object interactions from the package java.lang because it is the most heavily used package by nearly all of the training programs. Due to the unavailability of tools and corresponding results, we cannot quantitatively evaluate the coverage of object interactions of other multiple object protocol mining approaches currently [10, 11].

 To answer the question that whether our mined multiple object typestates describe typical API protocols, we have to evaluate the quality of mined typestates. To this end, we compare typestates of the same type mined from different applications. If the typestates appear in the results of at least two different applications, we can think that the typestates are not application specific but manifesting common API usage. We find that if two typestates models of the same type are mined from different applications and we do not consider interaction annotations, it is always the case that one is included in the other in that states and transitions of one typestate model is the subset of that of the other model, respectively. This is due to the fact that objects of the same type have the same abstract states under the same state abstraction function. Our miner can mine a model for each object created during the program execution. However, nearly all the benchmark programs create objects of some types that are never used by other benchmark programs. So we limit the inspected models to these ones that have to be mined from at least two benchmark programs. Because our major concern is object interactions, we choose to analyze state changes for transitions. Assume typestates *M*_1_ of type *t* and Γ as the set of all typestates models of type *t* mined from benchmarks different from that of *M*_1_. We consider a transition *e*_1_ of *M*_1_ is validated if there is one typestates model *M*_2_ that (1) *M*_2_ has a transition *e*_2_ that has the same source state, destination state, and method as that of *e*_1_, respectively, and (2) *e*_1_ and *e*_2_ have the same state change for each of interacting objects associated with the method of these two transitions. To measure the percentage of validated transitions of typestates, we compare the results from the benchmarks for the target packages together. The results are presented in Table 5. The results are very promising. Overall, most (84.0%) of transitions are validated. We conclude that most of mined typestates of multiple objects characterize common API usage instead of being incidental and application specific.

**Figure 3:**
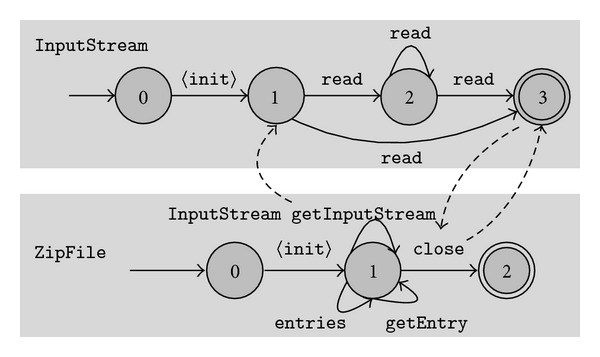
Mined typestates of ZipFile and InputStream.

We discuss another typestates model mined by our approach.  Figure 3 presents the typestates of ZipFile and InputStream. After an ZipFile object is constructed, several methods may be invoked, such as entries and getEntry. The getInputStream method returns an InputStream object. The dashed line from getInputStream to state 1 of InputStream indicates this interaction. Then, the method read is invoked to read bytes from this input stream. After finishing work, the method close is called to close the zip file. The dashed lines between close and state 3 of InputStream manifest that the close of the zip file also transitions the InputStream object to its final state. This captures the API protocol that close of ZipFile also closes the InputStream returned by getInputStream. Another interesting finding from Figure 3 is that close of ZipFile actually does not call close of InputStream. After inspecting the source code, we confirm this and find that ZipFile ensures the no usage of InputStream after its close call by a different way. The typestates of InputStream are extracted from objects of type ZipFileInputStream, that is, a private inner class of ZipFile.

### 4.3. Discussions

**Figure 4:**
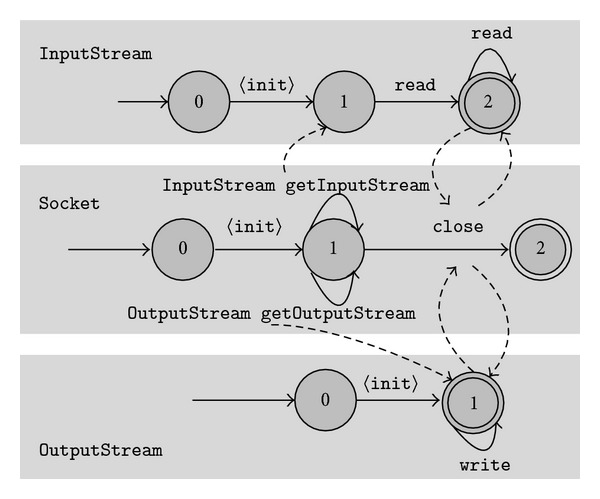
Mined typestates of Socket, InputStream and OutputStream.

In this section, we discuss several characteristics of our approach, mainly its drawbacks. Our approach is based on a simple state abstraction mechanism to label states. Although it is shown to be able to mine some useful single object typestates [14, 15], we find during our experiments that this state abstraction mechanism prevents us from mining some typical multiple object typestates. To compare our approach with other researcher's work [11], we mine several typestate models from traces of the conformance tests of the Apache Harmony project [26]. One of them is the Socket specification presented in Figure 4. The typestates capture the property that close of Socket also closes InputStream returned by getInputStream and OutputStream returned by getOutputStream. However, there are only two states in typestates of InputStream and only one state in typestates of OutputStream due to limitations of the used state abstraction (when counting the number of states, we do not consider the initial state, that is, the source state of a constructor). This permits read after InputStream is closed and write after OutputStream is closed which are illegal. At the same time, typestates in Figure 4 manifest a characteristic of our approach that some semantic unrelated events from different objects can be arbitrarily interleaved, which can enhance the completeness of mined typestates. For example, read of InputStream and write of OutputStream are separate from each other and can be arbitrarily interleaved because there are no direct or indirect interaction annotations between them. For approaches that take specification mining as a language learning problem from a set of input strings, it is not easy to capture this semantic unrelatedness without enough input samples.

There are approaches such as [27] that try to mine *deep* models. For the state abstraction, they do not simply map fields of reference types to *null* or *not null*, but also consider the fields of fields. However, considering unrelated fields can lead to unnecessary states that complicate the mined typestates. For example, states 1, 2, and 3 in the typestates of InputStream in Figure 4 are redundant to only represent the behavior of read. Because our general approach is modular in that different state labelling techniques can be integrated with it, new effective state abstraction mechanism can be employed to enhance mined typestates in future.

## 5. Related Work

An approach to mine single object typestates based on explicit object states is presented in [14, 15]. A typestate automaton for an object is a nondeterministic finite state automaton. Its states represent object states and are labeled with values of all fields of this object, and its transitions represent executions of methods of this object and are labeled with method names. A special state *ex* exists as the destination state of all method executions that throw an exception. For each method execution called on an object, there is a transition from the source state to the destination state. They combine all the transitions by merging states with the same field-value label to mine the typestates model of this object. The typestates automaton for a class consists of the union of all states and transitions of typestate automata of all its objects. Abstract states instead of concrete states are used in typestate automata. As states of typestate automata are usually anonymous, field-value labels of states are discarded, and states are identified by assigned abstract names. Our approach currently uses the same state abstraction function as [14, 15]. As we aim to mine normal behavior of programs, we do not consider method executions that throw exceptions and do not include a similar error state in our typestates. There are two main differences between their approach and ours. First, they cannot mine typestates of abstract types. Second, they can not mine interactions between objects; that is, they can only mine single object typestates. Dallmeier et al. [15] also present the techniques to systematically generate test cases that cover previously unobserved behavior to enrich mined single object typestates. Because the poor behavior coverage of traces is a common problem for all dynamic specification mining approaches, it is possible to adapt their test generation technique to enrich typestates of multiple objects.

Pradel and Gross [10] define the concept of *object collaborations* to capture related events based on the assumption that methods generally implement small and coherent pieces of functionality, and so the method invocations issued during a method's execution are related to each other. A collaboration is a sequence of method invocations associated with their receivers. To limit the number of events, they limit the depth of nested calls to a certain nesting level. Then, they apply several heuristics including the *package-based filtering* to filter out unrelated events. Similar object collaborations are grouped into a collaboration pattern. At last, an FSM is mined for each collaboration pattern. There are mainly two drawbacks of their approach. First, their approach can mine models for objects that are not interacting with each other when events of these objects are issued during a method's execution. Second, their approach may fail to group related events together when they exceed the scope of the execution of a method.

Lee et al. [11] present the *event specification* approach to capture related events based on the assumption that unit tests perform the behavior of tightly interacting objects, and so methods involved in a unit test likely obey some specification. An event specification is a set of methods together with a set of reference types, each of which is the type of a parameter or return of a method. Several heuristics including the *package-based filtering* are applied to further filter events involved in a unit test execution. At last, an event specification includes events that are directly or indirectly related. Two events are directly related if and only if they share at least one common receiver, method argument or method return, and related if and only if they are connected through a sequence of directly related events. Compared with their event specification, our interaction specification contains all public methods and all reference parameters and returns of these methods of a type. However, event specification does not ensure this. Their approach relies on the availability and quality of unit test cases. In addition, a unit test case may not contain complex interactions of many objects.

To maintain scalability, approaches to mine multiple object typestates based on predefined property templates can only mine models for simple property templates such as alternating templates over event pairs [4, 17] and resource usage patterns over event triples [28]. The predefined, simple property templates make learning an arbitrarily complex specification impossible. Gabel and Su [13] propose to learn simple generic patterns and compose them to construct large, complex specifications. They use two simple patterns *alternation* and *resource ownership* and two composition rules *branching* and *sequencing*. Their approach is shown to be able to capture most temporal specifications published in the literature. Our approach complements to theirs in that the interaction specifications of our approach are determined by the structure of the type, and they naturally capture all potential interactions.

Nguyen et al. [22] present a graph-based approach to mine the usage patterns of one or multiple objects from the source code. To model object usage, they present the graph-based representation called graph-based object usage model which includes action nodes of method calls, control nodes of control structure, and data flow among these nodes. They first extract object usage from the source code and then mine object usage patterns by identifying object usages with frequent appearance. Based on the observation that isomorphic graphs also contain isomorphic (sub)graphs, they mine the patterns increasingly by size (i.e., the number of nodes). Similarly to Pradel and Gross [10], their graph-based object usage models are extracted from individual methods, and the data flow analysis to determine the data dependency among nodes is intraprocedural and explicit. So their approach has the two drawbacks of Pradel and Gross [10] discussed above.

## 6. Conclusions

This paper presents a general multiple object typestates mining approach. We first mine single object typestates through state abstraction. These typestates are also annotated with abstract states of interacting objects to record object interactions. We then extract typestates for super types from typestates of their implementing classes. At the last step, different typestates are composed together to get typestates of multiple interacting objects. Our approach is scalable and useful in that it can mine typestates of typical API behavior with low learning complexity. However, the state abstraction mechanism used here is not very effective to mine multiple object typestates. In future, we plan to refine our approach by integrating new state labelling techniques.

## Figures and Tables

**Algorithm 1:**
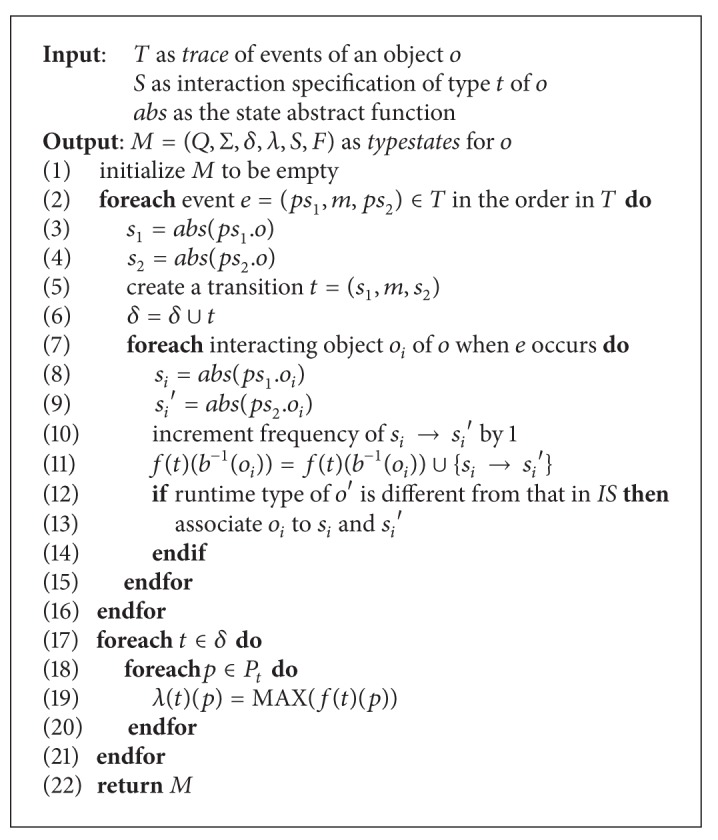
The algorithm to mine single object typestates annotated with interactions.

**Algorithm 2:**
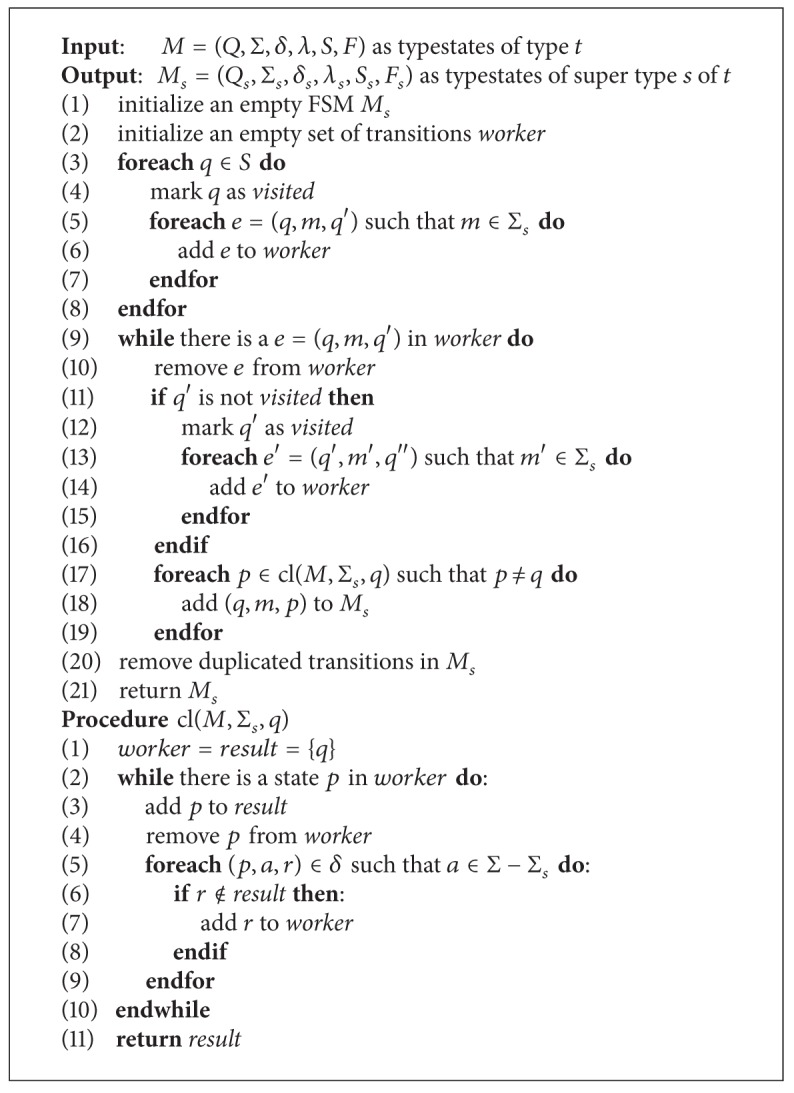
The algorithm to extract the typestates for a super type from the typestates of its implementing subtypes.

**Algorithm 3:**
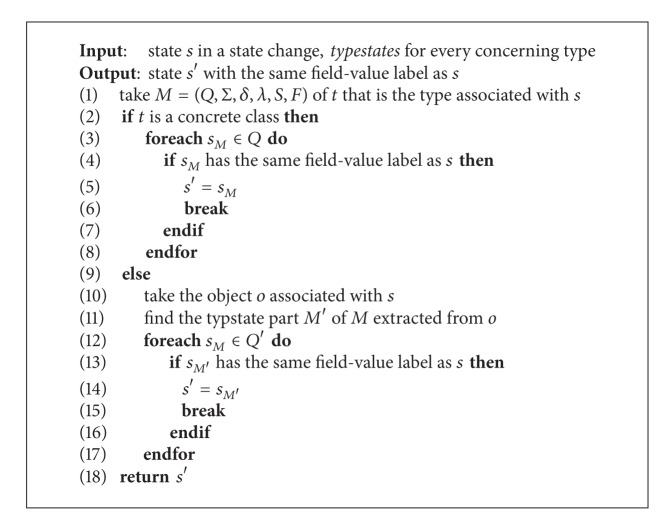
The algorithm to find the same state in corresponding typestates for a state in the state change of interaction annotations.

**Table 1:** Types of events and corresponding information traced by the tracing agent.

Event	Traced information
Method entry	Thread name, stack depth of this method, method name and signature, types and values for all parameters
Method exit	Thread name, stack depth of this method, method name and signature, type and value for return
Field modification	Type of class of object, type of declaring class of field, object tag, field name and type, new value

**Table 2:** Traces used in the experiments and analysis times in minutes.

Application	No. of events	Execution time	
		Single object typestate mining	Typestate extraction
antlr	8,079,678	6.5	1.2
bloat	7,235,945	7.5	1.6
chart	9,636,350	8	1.5
eclipse	5,348,521	3	0.7
fop	8,446,844	8.4	1.2
hsqldb	10,629,268	5	1.7
jython	812,978	0.5	0.2
luindex	7,196,849	9.5	0.4
lusearch	5,324,886	10	0.7
pmd	9,279,565	9.2	1.4
xalan	6,042,106	3.5	2.7

**Table 3:** Object interactions for target APIs. The third column is the number of types with no less than 10 interaction, and the last column is the average number of interactions per type.

Package	No. of interactions	No. of types (≥10)	Average number
java.lang	390	14	9.3
java.util	443	13	6.4
java.io	105	0	1.7

**Table 4:** Results of mined typestates. The third column is the total number of interactions for the package, and the last column is the number of mined typestates models with no less than 10 interactions.

Package	No. of single object typestates	No. of interactions	No. of models (≥10)
java.lang	27	161	3
java.util	32	77	0
java.io	30	23	0

**Table 5:** Quality of mined typestates.

Application	No. of transitions	No. of validated transitions	Percentage
antlr	384	329	85.7%
bloat	303	241	79.5%
chart	331	288	87.0%
eclipse	286	210	73.4%
fop	429	392	91.4%
hsqldb	408	331	81.1%
jython	335	264	78.8%
luindex	453	380	83.9%
lusearch	362	250	69.1%
pmd	402	398	99.0%
xalan	425	405	95.3%

Overall	4118	3488	84.0%
